# Discovery of IL-18 As a Novel Secreted Protein Contributing to Doxorubicin Resistance by Comparative Secretome Analysis of MCF-7 and MCF-7/Dox

**DOI:** 10.1371/journal.pone.0024684

**Published:** 2011-09-08

**Authors:** Ling Yao, Yan Zhang, Keying Chen, Xiaofang Hu, Lisa X. Xu

**Affiliations:** 1 School of Biomedical Engineering and Med-X Research Institute, Shanghai Jiao Tong University, Shanghai, China; 2 Shanghai Center for Systems Biomedicine, Shanghai Jiao Tong University, Ministry of Education, Shanghai, China; Enzo Life Sciences, Inc., United States of America

## Abstract

**Background:**

Resistance to chemotherapy is the major cause of failure in breast cancer treatment. Recent studies suggest that secreted proteins may play important roles in chemoresistance. We sought to systematically characterize secreted proteins associated with drug resistance, which may represent potential serum biomarkers or novel drug targets.

**Methodology/Principal Findings:**

In the present work, we adopted the proteomic strategy of one-dimensional gel electrophoresis followed by liquid chromatography-tandem mass spectrometry to compare the secretome of MCF-7 and doxorubicin-resistant MCF-7/Dox. A total of 2,084 proteins were identified with at least two unique peptides in the conditioned media of two cell lines. By quantification with label-free spectral counting, 89 differentially expressed secreted proteins (DESPs) between the two cell lines were found. Among them, 57 DESPs were first found to be related to doxorubicin resistance in this work, including 24 extracellular matrix related proteins, 2 cytokines and 31 unclassified proteins. We focused on 13 novel DESPs with confirmed roles in tumor metastasis. Among them, the elevated expression of IL-18 in doxorubicin-resistant cell lines and breast tumor tissues was validated and its role in doxorubicin resistance was further confirmed by cell viability experiments in the presence or absence of this protein.

**Conclusions/Significance:**

Comparative analysis of the secretome of MCF-7 and MCF-7/Dox identified novel secreted proteins related to chemotherapy resistance. IL-18 was further validated to contribute to doxorubicin resistance, in addition to its confirmed role in breast cancer metastasis. Due to its dual roles in both drug resistance and tumor metastasis, IL-18 may represent a useful drug target for breast cancer therapy.

## Introduction

Breast cancer is the most commonly diagnosed type of cancers among women. Chemotherapy is an important tool in the treatment of breast cancer. However, chemotherapy often fails due to drug resistance, especially multidrug resistance. Tumor cells are found to adopt multiple mechanisms to resist drugs, such as decreased uptake of drugs and/or enhanced efflux of drugs, altered metabolism of drugs, alterations in drug targets, activation of detoxify systems, enhanced DNA repair ability, inhibition of apoptosis [1]. These identified mechanisms are usually focused on the changes of membrane proteins and intracellular proteins in drug resistant tumor cells. The roles of secreted proteins in chemoresistance have not been clearly demonstrated.

Recently, several lines of evidence indicate that secreted proteins play critical roles in the acquisition of drug resistance. Extracellular matrix (ECM) components constitute a major part of secretome. It is widely reported that cancer cells become relatively resistant to the cytotoxic agents when cultured on ECM components, such as type IV collagen, laminin or fibronectin [2], [3]. The adhesion of cancer cells to ECM activates the integrin dependent pro-survival pathways to increase their drug resistance ability. Gene screening of drug sensitive and resistant cell lines also indicates that drug resistance is accompanied by the increased expression of ECM components. Staunton *et al.* (2001) generated gene expression-based classifiers to predict drug response for 232 compounds in 60 human cancer cell lines (the NCI-60) [4]. Strikingly, they discovered that gene expression-based classifiers for multiple drugs were significantly enriched for genes related to the cytoskeleton or ECM. For example, the 120-gene classifier for cytochalasin D resistance included 29 (24%) genes related to the cytoskeleton or ECM, such as FN1, COL6A1, COL4A1, COL4A2 and COL6A2. Besides ECM components, other secreted proteins are also found to contribute to drug resistance. Arlt *et al.* (2002) found that culture supernatants from pancreatic carcinoma drug resistant cell lines could induce the chemosensitive cells to acquire drug resistance [5]. Several secreted proteins have been identified to participate in the acquisition of chemoresistance, including some cytokines and growth factors. Interleukin-6 (IL-6) was found to cause multidrug resistance in breast cancer cells by activating the CCAAT enhancer-binding protein family of transcription factors and inducing *mdr1* gene expression [6]. Connective tissue growth factor (CTGF) conferred breast cancer cell drug resistance by augmenting a survival pathway through ERK1/2-dependent Bcl-xL/cIAP1 up-regulation [7]. In order to identify drug targets and potential serum biomarkers for clinical anti-cancer drug response prediction, a systematic screening of secreted proteins contributed to chemotherapy resistance is highly desired.

Proteomics provides a promising way to discover drug resistance related proteins. Both traditional two-dimensional gel electrophoresis and recently developed approaches, such as liquid chromatography-tandem mass spectrometry (LC-MS/MS), have been used to explore candidates involved in drug resistance [8], [9]. Recent emerging proteomic analysis of serum-free conditioned media (CM) of cell lines, termed as secretome analysis, provides a powerful way to identify secreted proteins related to drug resistance [10]–[12]. The strategy of one-dimensional gel electrophoresis in combination with LC-MS/MS (GeLC-MS/MS) was widely used in secretome studies [13], [14]. The pre-separation of proteins by SDS-PAGE to reduce sample complexity, followed by in-gel digestion and LC-MS/MS dramatically increase the analytical depth in complex samples. The label-free method is an effective way to semi-quantitate the abundance of detected proteins in different samples [15]. This method provides a higher dynamic range for quantification and more analytical depth than most stable isotope labeling techniques [16].

In the present work, we used the proteomic strategy of GeLC-MS/MS to compare the secretome between MCF-7 and its doxorubicin-resistant subcell line (MCF-7/Dox) to identify secreted proteins related to drug resistance. Doxorubicin is a DNA intercalating agent that produces free radicals and induces DNA double-strand breaks by interfering with DNA topoisomerases [17]. By using a proteomic strategy, we identified 1716 and 1261 proteins in the CM of MCF-7 and of MCF-7/Dox, respectively. Among the identified proteins, 244 were classified as secreted proteins. In addition, 835 proteins were predicted as potential secreted proteins by predictive software SignalP 3.0 and SecretomeP 2.0. We focused on 244 secreted proteins for further analysis. Based on label-free spectral counting, 89 differentially expressed secreted proteins (DESPs) between two cell lines were found. Among them, 57 DESPs have not been previously reported to be related to doxorubicin resistance. We further validated that interleukin-18 (IL-18) contributed to doxorubicin resistance. Considering the confirmed roles of IL-18 in breast cancer progression and metastasis, our work revealed for the first time to our knowledge that IL-18 might play dual functions in drug resistance and tumor metastasis.

## Results and Discussion

### MS Data Overview

**Figure 1:**
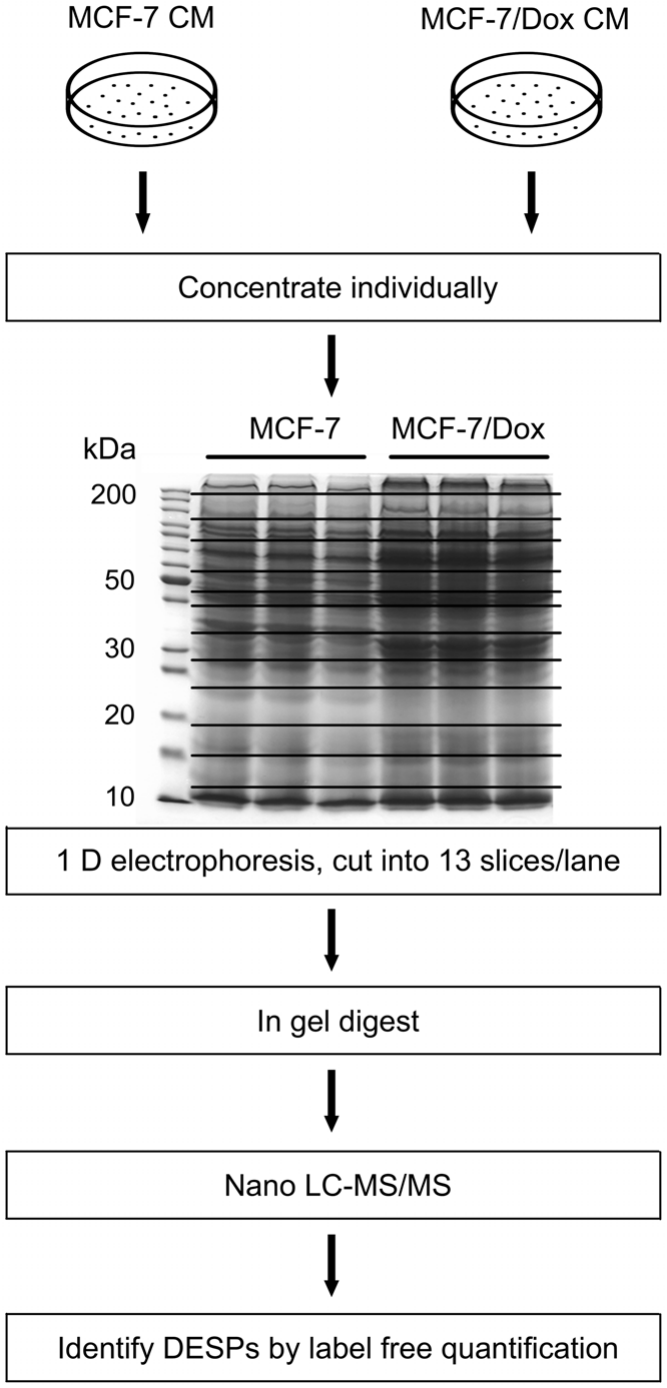
Workflow for the comparative secretome analysis of MCF-7 and MCF-7/Dox. Illustration of the label-free approach to identify the differential proteins in the CM of MCF-7 and MCF-7/Dox cell lines.

**Figure 2:**
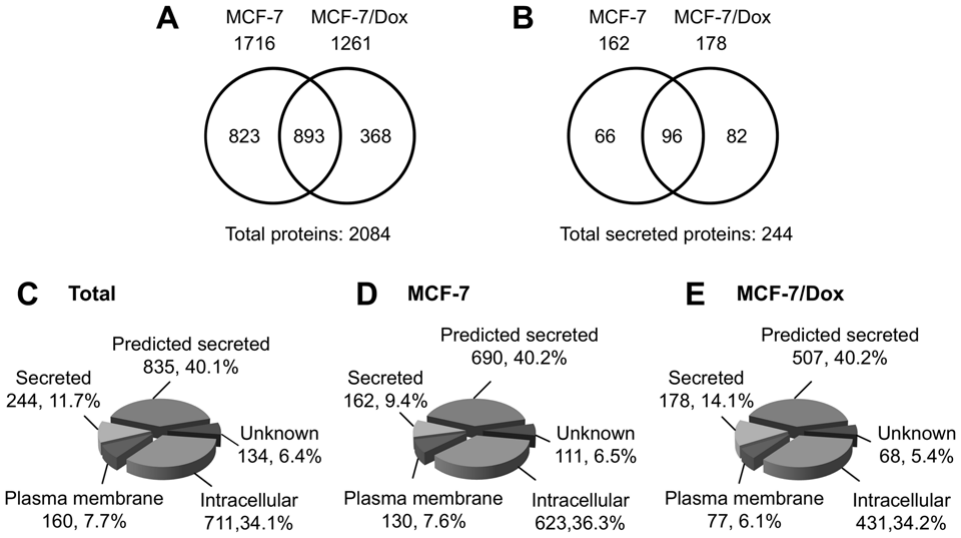
Overview of proteins identified in the CM of MCF-7 and MCF-7/Dox and their subcellular localization. Overlap of total proteins (A) and secreted proteins (B) identified in the CM of MCF-7 and MCF-7/Dox. (C) Subcellular localization of total proteins identified in the CM of MCF-7 and MCF-7/Dox. (D, E) Subcellular localization of proteins identified in the CM of MCF-7 and MCF-7/Dox, respectively.

As shown in Figure 1, the CM of MCF-7 and MCF-7/Dox were collected and analyzed by the proteomic strategy of GeLC-MS/MS. Three replicates of each cell line were analyzed. Proteins identified with at least two unique peptides were selected for further analysis. According to this criterion, a total of 2084 proteins were identified in the CM of two cell lines. As shown in Figure 2A, 893 proteins (42.9%) were detected in both cell lines, 823 and 368 proteins were only identified in CM of MCF-7 and MCF-7/Dox, respectively. For MCF-7/Dox, a large portion of proteins (893 in 1261, 70.8%) was shared with MCF-7.

We further analyzed the subcellular distribution of identified proteins. Among total 2084 identified proteins, 244 (11.7%) were classified as secreted proteins (Figure 2C). In addition, 835 (40.1%) proteins were predicted as potential secreted proteins by predictive software SignalP 3.0 and SecretomeP 2.0. Taken together, about 50% proteins were classified as secreted proteins or predicted secreted proteins. Among total 2084 identified proteins, we also detected 34.1% intracellular proteins. This was mainly due to the release of intracellular proteins from unavoidable dead cells during culture process. The proteins identified in the CM of MCF-7 or MCF-7/Dox showed similar subcellular distributions (Figure 2D, 2E).

We focused on 244 secreted proteins for further analysis. A total of 244 secreted proteins were identified in the CM of two cell lines, including 162 identified from MCF-7 and 178 identified from MCF-7/Dox. The overlap of secreted proteins in two cell lines was shown in Figure 2B: 96 secreted proteins were detected in both cell lines, while 66 and 82 secreted proteins were only detected in the CM of MCF-7 or MCF-7/Dox, respectively.

### Identification of Doxorubicin Resistance Related DESPs

Based on spectral counting of unique peptides, we used the Student's *t*-test to identify which of the 244 secreted proteins were differentially expressed in the two cell lines (p<0.01). We imposed an additional constraint for secreted proteins with abundance altered more than 2-fold. According to these criteria, we found 89 DESPs between two cell lines, with 61 up-regulated and 28 down-regulated in MCF-7/Dox.

**Figure 3:**
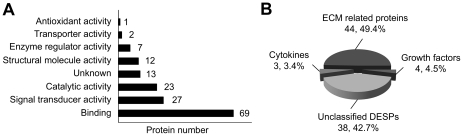
Functional analysis and classification of DESPs. (A) The functional analysis of DESPs between MCF-7 and MCF-7/Dox. (B) The classification of DESPs between MCF-7 and MCF-7/Dox.

The molecular functions of these 89 DESPs were classified according to Gene Ontology functional annotation. The top three functions were binding (69, 77.5%), followed by signal transducer activity (27, 30.3%) and catalytic activity (23, 25.8%) (Figure 3A). The molecular functions of 13 DESPs (14.6%) were still unknown.

### Classification of DESPs

To better understand the roles of these DESPs in the acquisition of drug resistance, we categorized the 89 DESPs into different classes, including ECM related proteins, growth factors and cytokines (Figure 3B). 51 DESPs were categorized into these groups (Table 1,2,3). The left 38 DESPs were grouped as unclassified DESPs (Table 4). The detailed information about the 89 DESPs was listed in supplementary Table S1.

**Table 1:** List of the ECM related DESPs.

Gene symbol	Spectral counting of peptides		P value	Rsc^a^	Ref^b^	Metastasis^c^
	MCF-7/Dox	MCF-7				
	Mean ± SD	Mean ± SD				
**ECM components**						
LAMB1	249.3±8.8	0.0±0.0	0.000	7.7	[20]	
CSPG4	152.0±21.5	0.0±0.0	0.001	7.0	[22]	yes
COL6A2	85.3±4.9	0.0±0.0	0.000	6.1	[20]	
COL12A1	66.0±8.5	0.0±0.0	0.000	5.8	[20]	
FBN2	68.3±13.1	0.0±0.0	0.002	5.8	[20]	
COL1A1	15.3±4.1	0.0±0.0	0.006	3.8		yes
FBN1	6.3±1.2	0.0±0.0	0.002	2.6	[20]	
LAMA1	578.0±14.4	0.7±0.9	0.000	8.3	[20]	
COL6A1	161.3±13.7	13.0±6.4	0.000	3.5	[20]	
LAMC1	470.0±36.6	116.0±5.4	0.000	2.0		
LAMA5	65.0±10.6	175.7±18.6	0.002	−1.4	[21], [22]	
COL18A1	1.7±2.4	71.3±20.8	0.009	−4.6		yes
COL12A1	0.0±0.0	218.3±31.4	0.001	−7.4		
**ECM remodeling related proteins**						
ADAMTS1	80.7±11.9	0.0±0.0	0.001	6.1	[29]	yes
CTHRC1	26.0±4.5	0.0±0.0	0.001	4.5		yes
EXTL2	21.0±5.0	0.0±0.0	0.004	4.2		
MMP1	2619.3±354.4	18.7±18.0	0.000	7.1	[23], [25], [26]	yes
TIMP2	166.7±31.9	32.0±10.8	0.005	2.4	[24]	
XYLT1	0.0±0.0	7.3±0.5	0.000	−2.8		
KLK6	0.0±0.0	34.0±8.0	0.004	−4.8		
PRSS23	0.0±0.0	72.3±12.7	0.001	−5.9		
Serpin Family						
SERPINB2	142.3±26.6	0.0±0.0	0.002	6.9	[23]	
SERPINE2	90.7±8.7	0.0±0.0	0.000	6.2	[28]	yes
SERPING1	13.7±2.9	0.0±0.0	0.003	3.6		
SERPINE1	824.7±125.9	1.0±1.4	0.001	8.6	[23]	yes
SERPINA5	26.7±2.5	11.7±0.5	0.001	1.1		
SERPINA3	0.0±0.0	177.7±28.4	0.001	−7.1		
**Other ECM related proteins**						
SPARC	532.3±29.4	0.0±0.0	0.000	8.8	[21]	yes
NID2	223.3±9.9	0.0±0.0	0.000	7.5		
MATN2	118.3±4.2	0.0±0.0	0.000	6.6		
LUM	43.7±9.2	0.0±0.0	0.003	5.2		yes
MFGE8	33.7±9.0	0.0±0.0	0.006	4.8	[28]	
CCDC80	35.3±3.3	0.0±0.0	0.000	4.9		
ICAM1	18.7±0.9	0.0±0.0	0.000	4.0	[42]	yes
NID1	6.0±0.8	0.0±0.0	0.000	2.6		
ECM1	223.3±11.0	1.3±1.9	0.000	6.5	[20], [21]	yes
COCH	132.7±5.0	8.7±2.6	0.000	3.8		
LGALS3BP	561.0±22.8	81.0±16.3	0.000	2.8	[44]	yes
THBS1	404.0±23.4	1634.3±289.4	0.004	−2.0		yes
AGRN	83.7±14.1	360.0±13.1	0.000	−2.1		yes
MATN2	0.0±0.0	17.7±3.3	0.002	−3.9		
PXDN	0.0±0.0	103.7±16.9	0.001	−6.4		
HMCN1	0.0±0.0	380.3±94.3	0.005	−8.2		
FREM2	0.0±0.0	333.0±19.3	0.000	−8.0		

a R_SC_: the spectral count fold-change ratio for protein between MCF-7/Dox and MCF-7. It was calculated according to Eq (1).

b references for doxorubicin resistance.

c “yes” indicates the same expression tendency between drug resistance and tumor metastasis.

**Table 2:** List of growth factors among the DESPs.

Gene symbol	Spectral counting of peptides		P value	Rsc^a^	Ref^b^	Metastasis^c^
	MCF-7/Dox	MCF-7				
	Mean ± SD	Mean ± SD				
CTGF	172.0±14.9	0.0±0.0	0.000	7.1	[7], [29]	yes
CYR61	152.3±10.9	0.0±0.0	0.000	7.0	[45]	yes
NOV	50.3±2.6	0.0±0.0	0.000	5.4	[46]	yes
DKK1	18.0±5.1	0.0±0.0	0.008	4.0	[23]	yes

a R_SC_: the spectral count fold-change ratio for protein between MCF-7/Dox and MCF-7. It was calculated according to Eq (1).

b references for doxorubicin resistance.

c “yes” indicates the same expression tendency between drug resistance and tumor metastasis.

**Table 3:** List of cytokines among the DESPs.

Gene symbol	Spectral counting of peptides		P value	Rsc^a^	Ref^b^	Metastasis^c^
	MCF-7/Dox	MCF-7				
	Mean ± SD	Mean ± SD				
IL-6	68.0±10.7	0.0±0.0	0.001	5.8	[6]	yes
IL-18	11.3±1.9	0.0±0.0	0.001	3.4		yes
ILEI	31.0±2.2	6.3±5.3	0.004	2.1		yes

a R_SC_: the spectral count fold-change ratio for protein between MCF-7/Dox and MCF-7. It was calculated according to Eq (1).

b references for doxorubicin resistance.

c “yes” indicates the same expression tendency between drug resistance and tumor metastasis.

**Table 4:** List of the remained DESPs.

Gene symbol	Spectral counting of peptides		P value	Rsc^a^	Ref^b^	Metastasis^c^
	MCF-7/Dox	MCF-7				
	Mean ± SD	Mean ± SD				
PTGDS	193.3±37.1	0.0±0.0	0.002	7.3		
MSLN	180.0±31.9	0.0±0.0	0.001	7.2	[26]	yes
LOXL2	83.3±6.8	0.0±0.0	0.000	6.1		yes
TNFRSF6B	42.7±1.2	0.0±0.0	0.000	5.2	[41]	yes
AEBP1	42.0±4.3	0.0±0.0	0.000	5.1		
EPDR1	39.7±5.4	0.0±0.0	0.000	5.1		
CDA	32.0±6.7	0.0±0.0	0.002	4.8		
IGFBP6	31.0±2.9	0.0±0.0	0.000	4.7		
TWSG1	20.7±1.7	0.0±0.0	0.000	4.2		
IGFBPL1	15.3±1.7	0.0±0.0	0.000	3.8		
CPA4	14.7±2.5	0.0±0.0	0.001	3.7		
MUC16	8.0±1.4	0.0±0.0	0.001	2.9	[43]	yes
FSTL1	255.3±27.2	0.7±0.9	0.000	7.1		
C1R	107.7±11.9	0.7±0.9	0.000	5.9		
SEMA3D	91.0±2.2	0.7±0.9	0.000	5.6		
PTX3	260.3±33.8	2.7±3.8	0.000	6.1	[23]	
PLA2G15	47.7±7.6	2.0±2.8	0.001	3.9		
ERAP1	16.0±3.6	1.0±1.4	0.005	3.0		
NPC2	111.7±24.6	9.3±8.2	0.005	3.4		
RNASE4	47.7±2.1	4.7±4.6	0.000	3.1		
VCL	333.7±11.1	48.7±7.6	0.000	2.8		
PSAP	77.7±9.0	12.0±0.8	0.001	2.6	[22]	yes
TGFBI	142.7±22.2	23.3±4.5	0.002	2.6		yes
TFRC	56.7±9.5	162.3±15.8	0.001	−1.5	[30]	
RNPEP	24.3±6.1	79.7±11.6	0.004	−1.6		
CPE	6.3±0.5	24.3±3.7	0.002	−1.7		
CLU	20.3±1.7	164.7±5.7	0.000	−2.9		
RNASET2	7.3±1.2	62.7±14.6	0.006	−2.9		yes
GSN	16.0±2.2	195.3±16.8	0.000	−3.5	[21], [22]	yes
SEMA3C	3.3±1.2	272.0±49.4	0.002	−5.9		
NTN1	0.0±0.0	12.3±2.1	0.001	−3.4		
NUCB2	0.0±0.0	14.0±3.7	0.006	−3.6		
IDE	0.0±0.0	16.3±2.4	0.001	−3.8		yes
FLRT3	0.0±0.0	17.7±3.4	0.002	−3.9		
METRNL	0.0±0.0	23.3±4.5	0.002	−4.3		
PDGFRL	0.0±0.0	26.7±7.7	0.008	−4.5		yes
STC1	0.0±0.0	40.3±4.0	0.000	−5.0		
IGFBP2	0.0±0.0	127.7±12.9	0.000	−6.7		

a R_SC_: the spectral count fold-change ratio for protein between MCF-7/Dox and MCF-7. It was calculated according to Eq (1).

b references for doxorubicin resistance.

c “yes” indicates the same expression tendency between drug resistance and tumor metastasis.

The largest portion of the DESPs were ECM related proteins (44, 49.4%), including 13 ECM components, 14 ECM remodeling related proteins and 17 proteins which can interact with the ECM components or contribute to the ECM structure (Table 1). This finding was consistent with the widely reported roles of ECM in drug resistance. We found most of the ECM related DESPs (31 of 44, 70.5%) were up-regulated in MCF-7/Dox. We noticed that among 14 ECM remodeling related proteins, 6 proteins belonged to the serpin family, including SERPINB2, SERPINE2, SERPING1, SERPINE1, SERPINA5 and SERPINA3. The serpins are the largest superfamily of serine protease inhibitors, and are involved in diverse biological processes, including ECM maintenance and remodeling, tumor cell invasion, and inflammation [18].

Among the identified DESPs, there were four growth factors, including CYR61, CTGF, NOV and DKK1 (Table 2). All these growth factors were only detected in the CM of MCF-7/Dox. Notably, three growth factors (CYR61, CTGF, NOV) belong to the same family, namely CCN family [19]. In addition, the cytokines IL-6, IL-18 and ILEI (FAM3C) were also up-regulated in MCF-7/Dox (Table 3).

### Novel Identified Doxorubicin Resistance Related Secreted Proteins

The DESPs found in this work were extensively compared with previous studies to identify novel chemoresistance relevant secreted proteins. The reported proteins were marked in Table 1,2,3,4.

First, we compared DESPs with doxorubicin resistance related genes which were found by functional genomics studies [4], [20]–[27]. For example, Iseri *et al.* (2009) demonstrated the alterations in gene expression levels of ECM proteins in doxorubicin-resistant MCF-7 cells [20]. Among those proteins, 8 of them coincided with our identified DESPs, including LAMB1, COL6A2, COL12A1, FBN2, FBN1, LAMA1, COL6A1 and ECM1. Among the gene expression signature for doxorubicin resistance based on the NCI-60 cell line panel [4], [21], [22], we found 6 proteins overlapped with our identified DESPs, including CSPG4, LAMA5, ECM1, SPARC, GSN and PSAP. Then we considered doxorubicin resistance related genes identified in clinical samples of breast cancer [28]–[34]. We found 4 proteins showed similar up-/down-regulation tendency with our DESPs, including MFGE8, SERPINE2, ADAMTS1 and CTGF. Combining these results, a total of 25 DESPs were previously identified by transcriptional profiling studies.

Second, we compared our DESPs with doxorubicin resistance related proteins identified by proteomic studies [8], [35]–[40]. Although differentially expressed proteins between MCF-7 and its doxorubicin resistant cell were extensively studied by proteomic methods, the secretome of two cell lines has not been compared. Among those works, we found no protein overlapped with our DESPs.

To avoid omission of doxorubicin resistance related proteins which were identified by other studies, we searched against PubMed with keywords of “gene/protein name, drug resistance and adriamycin/doxorubicin” for each of the remaining DESPs. This analysis identified additional 7 reported DESPs, including TNFRSF6B [41], ICAM1 [42], MUC16 [43], LGALS3BP [44], CYR61 [45], NOV [46] and IL-6 [6].

Taken together, among 89 DESPs, 32 have been reported in previous studies, leaving 57 DESPs as novel identified adriamycin/doxorubicin resistance related secreted proteins. These 57 novel identified proteins included 24 ECM related proteins, 2 cytokines and 31 unclassified proteins.

### Secreted Proteins with Potential Dual Functions in Drug Resistance and Tumor Metastasis

The drug resistance and metastasis of tumor cells have often been considered and studied as independent events. However, recent studies indicate that the acquisition of these two abilities are tightly related. Carcinostatic agents may increase the number and the growth rate of metastases, which are often more drug resistant than their primary tumors [47], [48]. The drug resistant cancer cells were found to be more metastatic than their parental cell lines [49], [50]. Several proteins with dual functions in drug resistance and tumor metastasis have been found in recent years. The proteins with dual functions are ideal cancer therapeutic targets. To identify the potential dual functional proteins, 89 DESPs were searched against PubMed to confirm their roles in tumor metastasis. We found that 32 DESPs were reported to contribute to tumor metastasis with the same up- or down-regulation tendency in drug resistance. As mentioned above, 89 DESPs identified in this work included 32 drug resistance related proteins reported in previous work and 57 novel identified proteins. Among 32 reported drug resistance related secreted proteins, 19 were found to participate in tumor metastasis. Among 57 novel identified drug resistance related secreted proteins, 13 were found to contribute to tumor metastasis. These 13 proteins have the potential to be validated as novel dual functional proteins.

We noticed that four growth factors all have confirmed dual functions in chemoresistance and cancer metastasis. Interestingly, three growth factors (CYR61, CTGF, NOV) belong to the CCN family. The CCN family consists of six members: CTGF, NOV, CYR61, WISP-1, WISP-2, and WISP-3 [19]. Their expression can promote cellular proliferation, migration, adhesion, and extracellular matrix formation, as well as the regulation of angiogenesis and tumorigenesis [7], [19]. The function of each member of CYR61, CTGF, NOV in drug resistance or cancer metastasis has been reported separately [7], [45], [46], [51]–[53]. The activation of some critical pathways by these growth factors contributes to the acquisition of dual functions in drug resistance and cancer metastasis. CTGF activates integrin α_v_β_3_-dependent ERK1/2 pathway to promote the migration and drug resistance of breast cancer cells [7], [54]. Likewise, CYR61 contributes to breast cancer cell drug resistance via activation of the integrin α_v_β_3_/NF-κB/XIAP signaling pathway [45], and it promotes tumor cell motility/invasion by an integrin α_v_β_3_/NF-κB-dependent COX-2 up-regulation pathway [55].

Noteworthy, all three cytokines in our identified DESPs have confirmed functions in breast cancer metastasis. Among them, only IL-6 has confirmed function in drug resistance. It has been reported that IL-6 can confer multiple cancer cells resistance to chemotherapeutic compounds, such as myeloma [56], prostate cancer [57], ovarian cancer [58] and breast cancer cells [6]. The monoclonal antibody of IL-6 has been used as second-line therapy for men with castration-resistant prostate cancer [59]. However, the roles of IL-18 and ILEI in drug resistance have not been demonstrated before.

**Figure 4:**
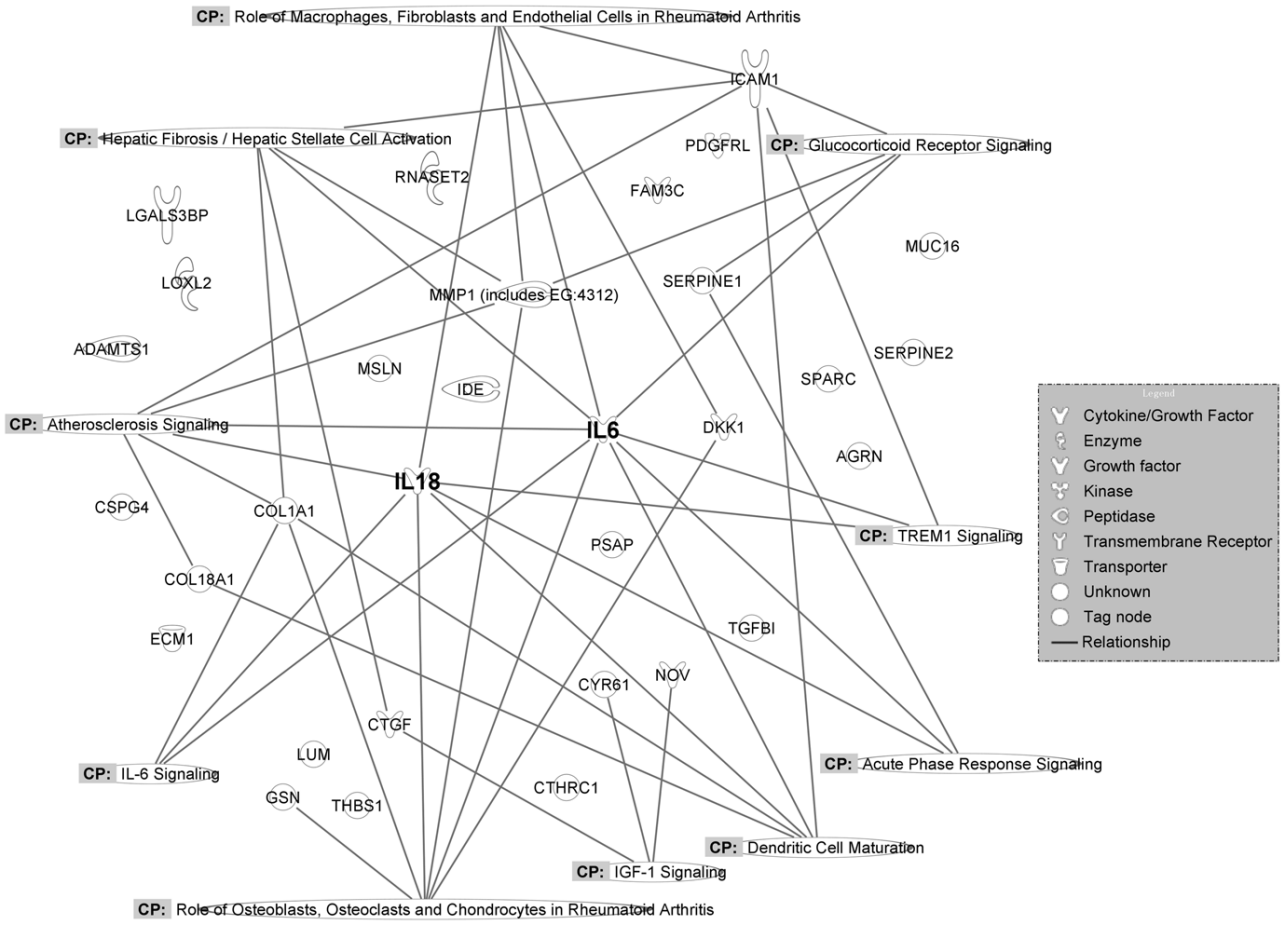
Canonical pathways analysis of DESPs with potential dual functions in drug resistance and tumor metastasis. 32 DESPs with dual functions were analyzed by software IPA. The top 10 canonical pathways were mapped onto the DESPs, such as IGF-1 signaling, acute phase response signaling and IL-6 signaling etc. IL-6 and IL-18 were located as centers to link multiple signaling pathways.

We used the Ingenuity Pathways Analysis (IPA) software to demonstrate the networks of the total 32 DESPs with potential dual functions (Figure 4). It was obvious that IL-6 and IL-18 were located as centers to link multiple signaling pathways. IL-18 participated in 7 pathways among the top 10 canonical pathways, including IL-6 signaling pathway, role of macrophages, fibroblasts and endothelial cells in rheumatoid arthritis pathway, and atherosclerosis signaling pathway. These signaling pathways are involved in cellular proliferation, cellular apoptosis, ECM remodeling, etc., suggesting that IL-18 has the potential to contribute to drug resistance. However, the role of IL-18 in drug resistance has not been validated.

### Validation the Role of IL-18 in Drug Resistance

IL-18 was discovered as an interferon-γ-inducing factor [60]. It belongs to IL-1 cytokine superfamily [61]. Except its functions in inflammatory and immune response, it can promote tumor progression via promoting angiogenesis, promoting tumor growth and metastasis, and escaping immune response [62]. IL-18 can promote the metastasis of multiple cancers, such as breast cancer [63], [64], myeloma [65], gastric cancer [66] and leukemia [67]. The blockade of IL-18 bioactivity can inhibit the lung metastasis of 4T1 breast cancer in mouse model [68]. Clinical data indicate that the expression level of IL-18 is increased in cancer patients, and correlated with tumor size, clinical stage, lymph node metastasis, distant metastasis and prognosis [64]. These evidences indicate that IL-18 is an important cytokine to promote cancer progression and metastasis.

**Figure 5:**
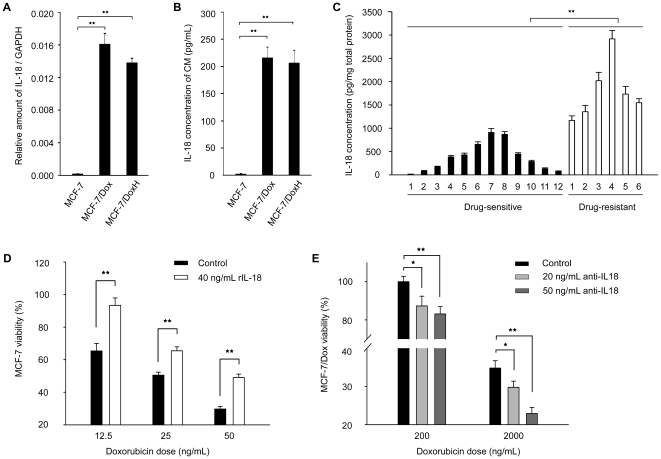
Validation of the expression level of IL-18 and its role in drug resistance. (A) The mRNA level of IL-18 in MCF-7, MCF-7/Dox and MCF-7/DoxH. The amount of IL-18 mRNA was normalized against the expression of GAPDH and the data were presented as mean ± SD (n = 3). **, p<0.01. (B) The concentration of IL-18 in the CM of MCF-7, MCF-7/Dox and MCF-7/DoxH. The data were presented as mean ± SD (n = 3). **, p<0.01. (C) The expression level of IL-18 in the lysates of 18 breast tumor tissues, including 12 drug-sensitive tissues and 6 drug-resistant tissues. The data were presented as mean ± SD (n = 3). **, p<0.01. (D) The doxorubicin dose dependent survival rate of MCF-7 in the absence or presence of 40 ng/mL rIL-18. Data were presented as mean ± SD (n = 3). **, p<0.01. (E) The effect of anti-IL-18 neutralization on the survival rate of MCF-7/Dox. Data were presented as mean ± SD (n = 3). *, p<0.05, **, p<0.01.

We first validated the elevated expression level of IL-18 in doxorubicin resistant cell lines. Besides MCF-7 and MCF-7/Dox, another MCF-7 subcell line with higher resistance ability to doxorubicin (MCF-7/DoxH) was also included in the study. As shown in Figure 5A, the mRNA level of IL-18 was significantly increased in MCF-7/Dox (91.9±10.6-fold) or MCF-7/DoxH (78.8±5.5-fold) compared to MCF-7. We also determined the IL-18 concentration in the cell lysates and CM in three breast cancer cell lines. Our results showed that in cell lysates, the concentration of IL-18 was dramatically increased from 33.4±5.6 pg/mg total protein for MCF-7 to 9430.5±270.5 pg/mg total protein for MCF-7/Dox and 9142.4±524.6 pg/mg total protein for MCF-7/DoxH. Accordingly, the secreted IL-18 in the CM was increased from undetectable for MCF-7 to 215.8±19.6 pg/ml for MCF-7/Dox and 206.4±23.2 pg/ml for MCF-7/DoxH (Figure 5B). It is evident that there is a significant increase in the expression of IL-18 in two drug resistant cell lines at both mRNA and protein level. Interestingly, the induction of IL-18 expression was similar in MCF-7/Dox and MCF-7/DoxH without further elevation with the increased drug resistance. Considering cytokines, including IL-18, usually exert biological functions at concentrations as low as picomolar levels [64], the expression level of IL-18 in MCF-7/Dox and MCF-7/DoxH may be high enough to exert its function in doxorubicin resistance. On the other hand, drug resistance is a complex event involving the changes of hundreds of genes/proteins which accounting for multiple mechanisms [1], [4], [27]. Besides the contribution of IL-18 in doxorubicin resistance, the acquisition of higher doxorubicin resistance ability in MCF-7/DoxH may require the involvement of other proteins and mechanisms.

To test if IL-18 plays an important role in drug resistance in breast cancer patients, we further determined the concentration of IL-18 in the tumor tissue lysates of 18 breast cancer patients received neoadjuvant chemotherapy before surgery (Figure 5C). In 12 drug-sensitive tumor lysates, the average concentration of IL-18 was 371.6 pg/mg total protein, ranging from 13.5±3.1 to 910.6±85.1 pg/mg total protein. Notably, the concentration of IL-18 was dramatically increased in 6 drug-resistant tumor lysates, the average concentration of IL-18 was 1790.3 pg/mg total protein, ranging from 1168.4±97.5 to 2917.3±180.8 pg/mg total protein. All of these results indicated that IL-18 played an important role in drug resistance.

The role of IL-18 in doxorubicin resistance was further validated by the increased doxorubicin resistance of MCF-7 in the presence of recombinant human IL-18 (rIL-18) and decreased doxorubicin resistance of MCF-7/Dox with the neutralization of IL-18. As shown in Figure 5D, in the presence of rIL-18, the survival rate of MCF-7 was significantly increased at doxorubicin concentration of 12.5, 25.0, 50.0 ng/mL. For example, at doxorubicin concentration of 12.5 ng/mL, the survival rate of MCF-7 was increased from 65.4%±4.6% to 93.4%±4.6% (p<0.01) in the presence of rIL-18. The fifty percent inhibition of cell proliferation (IC_50_) of MCF-7 for doxorubicin was elevated from 24.9 ng/mL to 45.5 ng/mL with the existence of 40.0 ng/mL rIL-18 in culture medium (Figure S2A). We also tested the effect of neutralization of IL-18 on the survival of MCF-7/Dox. As shown in Figure 5E, at the doxorubicin concentration of 200 ng/mL, the cell viability was decreased from 100%±2.8% to 87.3%±5.1% (p<0.05) and 83.1%±3.9% (p<0.01) with the existence of 20 and 50 ng/mL anti-IL-18 antibody, respectively. At the doxorubicin concentration of 2000 ng/mL, the cell viability was decreased from 34.8%±1.9% to 29.8%±1.6% (p<0.05) and 22.9%±1.6% (p<0.01) with the existence of 20 and 50 ng/mL anti-IL-18 antibody, respectively. The viability of MCF-7/Dox was also decreased at other doxorubicin concentrations in a manner which is dependent on the concentration of anti-IL-18 antibody (Figure S2B). Our results demonstrated that IL-18 also contributed to drug resistance besides its roles in promoting breast cancer metastasis. As a secreted protein with dual functions, IL-18 could be an ideal drug target of breast cancer therapy.

Drug resistance and tumor metastasis are the major causes of chemotherapy failure and mortality in breast cancer patient. The identification of proteins with dual functions in drug resistance and tumor metastasis is critical for understanding the linkage between these two processes and designing better drugs and therapeutic strategies. As ideal drug targets, the discovery of secreted proteins with dual functions is highly desired. In this work, we presented 13 secreted proteins with potential dual functions and demonstrated for the first time to our knowledge that IL-18 might play dual functions in drug resistance and breast cancer metastasis.

### Conclusion

In the present work, we compared the secretome of MCF-7/Dox and MCF-7 to identify doxorubicin resistance related secreted proteins. We found 89 DESPs between the two cell lines. Noteworthy 57 DESPs were first found to be related to doxorubicin resistance in this work. Among them, 13 DESPs have been reported to participate in tumor metastasis. These 13 DESPs were potential novel dual-functional proteins, with confirmed role in tumor metastasis and unvalidated role in drug resistance. One of them, IL-18, was further validated to contribute to doxorubicin resistance. As a newly identified protein with dual functions in drug resistance and breast cancer metastasis, IL-18 is a promising target for cancer therapy.

## Materials and Methods

### Ethics Statement

Human breast cancer tissue samples were obtained from the Ruijin Hospital, Shanghai Jiao Tong University, upon the approval of the Scientific and Ethical Committee of Shanghai Jiao Tong University. The written informed consent was obtained from all patients. The study was conducted according to the principle of the Helsinki Declaration. Data were analyzed anonymously.

### Cell lines, tumor tissues and reagents

The breast cancer cell line MCF-7 was purchased from the American Type Culture Collection. MCF-7/Dox and MCF-7/DoxH were purchased from Nanjing KeyGen Biotech. Co. Ltd, China. MCF-7/Dox and MCF-7/DoxH were developed from sensitive MCF-7 by stepwise selection in increasing concentrations of doxorubicin. MCF-7/Dox and MCF-7/DoxH showed 35-fold, 265-fold increase in doxorubicin resistance compared to MCF-7, respectively (Figure S1A and S1B). The method of the drug resistant characteristic assay of MCF-7/Dox and MCF-7/DoxH is described in Text S1. The 18 post-chemotherapy breast tumor tissue samples were obtained from patients received four cycles of cyclophosphamide - doxorubicin - 5-fluorouracil before surgery. Responses to chemotherapy were classified according to the UICC criteria [69]. Partial response (PR) was defined as a reduction of at least 50% in measurable tumor lesions and no new lesions appears, progressive disease (PD) was defined as a 25% or more increase in the measurable lesions or the appearance of new tumor lesions. We defined the tumors with response PR as drug-sensitive samples and the tumors with response PD as drug-resistant samples. The information of patients was shown in supplementary Table S2. RPMI medium 1640 was purchased from Gibco. Doxorubicin was purchased from Zhejiang Hisun Pharmaceutical Co., China. All other chemicals, unless indicated otherwise in the text, were from Sigma-Aldrich. The highest available grades were used throughout the study.

### Cell Culture

MCF-7 cell line was cultured in RPMI medium 1640 supplemented with 10% fetal bovine serum, penicillin (40 kU/L) and streptomycin (40 mg/L) at 37°C in 5% CO_2_. MCF-7/Dox and MCF-7/DoxH were cultured in the same medium with additional supplement of 200 ng/mL and 1000 ng/mL doxorubicin, respectively.

### CM Collection and Concentration

When cells were grown to approximately 75% confluence in 10 cm culture dishes, the culture medium was changed to serum free conditioned medium (CM). As described previously [70], we optimized the cell wash procedure to avoid the contamination of remained bovine serum. The cells were rinsed with serum free medium at 37°C for 15 min twice and then 60 min twice. Then the cells were incubated in the serum free medium at 37°C for 24 h. The cell death rate was usually under 3%. The CM of each cell line was collected, centrifuged at 1,500 rpm for 10 min, filtrated by 0.22 µm filter, and then added protease inhibitor cocktail (Roche, Mannheim, Germany) before being stored at −80°C.

Three replicates of CM from 8.0×10^6^ cells for each cell line were concentrated by the Ultra-15 centrifugal filter devices with a 3 kDa cutoff (Millipore, Bedford, MA). The protein concentration of concentrated samples was determined by Bradford assay. Then these samples were lyophilized for further analysis.

### One-dimensional Gel Electrophoresis and In-gel Digestion

Three replicates of concentrated CM for each cell line were separated on the 12% SDS-PAGE gel, and stained with Coomassie Blue. After extensive decolorization, each gel lane was excised into 13 sections. Each excised section was cut into approximately 1 mm cubes and destained by incubation in 50% acetonitrile in 50 mM ammonium bicarbonate. After destained, the gel pieces were reduced by incubation in a solution of 50 mM tris (2-carboxyethyl) phosphine in 25 mM ammonium bicarbonate at 60°C for 10 min. For alkylation of proteins, the gel was incubated in a solution of 100 mM iodoacetamide at room temperature for 60 min, followed by washing the sample using 50% acetonitrile in 50 mM ammonium bicarbonate for three times. After dehydrated in 100% acetonitrile for 15 min, gel pieces were completely dried by SpeedVac. Then the gel pieces were swollen in 50 µL of 25 mM ammonium bicarbonate buffer containing 0.01 µg/µL trypsin (Promega, Madison, WI) and incubated overnight at 37°C. Peptides were extracted with 50% acetonitrile containing 5% formic acid four times, dried by vacuum centrifugation at 60°C, and stored at −20°C for further analysis.

### Nano LC-MS/MS

The tryptic peptide digests of the proteins were analyzed using an MDLC system (Michrom Bioresources Inc., Auburn, CA) coupled with a Thermo Finnigan 2-D linear ion trap mass spectrometer (LTQ^XL^, Thermo Inc., San Jose, CA). Each peptide sample was re-dissolved in 5% acetonitrile with 0.1% formic acid, and then loaded onto a Peptide Captrap column (Michrom Bioresources Inc., Auburn, CA) with the autosampler of the MDLC system. To desalt and concentrate the sample, the trap column was washed with 5% acetonitrile with 0.1% formic acid at a flow rate of 10 µL/min for 10 min. Then trapped peptides were released and separated on a C18 capillary column (0.1 mm i.d.×150 mm, 3 µm, 200 Å, Michrom Bioresources Inc., Auburn, CA). The peptides were separated using a solvent system with solvent A consisting of 99.9% water and 0.1% formic acid, and solvent B consisting of 99.9% acetonitrile and 0.1% formic acid. The peptides were eluted with linear gradient from 5% B to 35% B in 120 min with a constant flow rate of 500 nL/min. The LC setup was coupled online to a LTQ using a nano-ESI source (ADVANCE, Michrom Bioresources Inc., Auburn, CA) in data-dependent acquisition mode (m/z 400–1800). The temperature of heated capillary was set at 200°C and spray voltage was 1.2 kV. The mass spectrometer was set as one full MS scan followed by ten MS/MS scans on the ten most intense ions from the MS spectrum with the following dynamic exclusion settings: repeat count = 2, repeat duration = 15 s, exclusion duration = 30 s.

### Protein Identification

All data files were created by searching MS/MS spectra against the Human International Protein Index protein sequence database (IPI.Human.v3.63.fasta, 84118 entries), by using the TurboSEQUEST program in the BioWorks 3.3 software suite, with a precursor-ion mass tolerance of 2.0 amu and fragment-ion mass tolerance of 1.0 amu. Trypsin was set as the protease with two missed cleavage sites allowed. Carbamidomethylation (+57.02150 Da) was searched as a fixed modification on cysteine, representing alkylation with iodoacetamide, while oxidized methionine (+15.99492 Da) was searched as a variable modification. The searched peptides and proteins were validated by PeptideProphet and ProteinProphet in the Trans-Proteomic Pipeline (TPP, v.4.2) using default parameters. Proteins with ProteinProphet P value greater than 0.9 and with no less than two kinds of unique peptides were considered as true identifications. A randomized database of the IPI.Human.v3.63.fasta was used as a decoy database to calculate the false discovery rate (FDR) of protein identification. The FDR was calculated by the ratio of the number of matches to the randomized database to the combined number of matches to the IPI.Human.v3.63.fasta and its randomized derivative. FDR for ProteinProphet P≥0.9 was less than 1%. Proteins containing the same peptides were grouped, and only one protein with highest probability in each group was remained.

### Bioinformatics Analysis

The proteins identified with at least two unique peptides were selected for further analysis. Spectral counts of unique peptides were used for protein quantification. The data were further analyzed with SPSS 14.0 software. Student's *t*-test was used to select data sets from two groups with statistical significance. To compare the relative abundance of each protein between MCF-7/Dox and MCF-7, we calculate the spectral count fold-change ratios (R_SC_) using following algorithm [15], [71]:
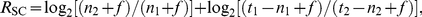
(1)where for each protein, R_SC_ is the log_2_ ratio of protein abundance between MCF-7/Dox and MCF-7; *n*_1_ and *n*_2_ are the average of spectral counts of this protein in three replicates for MCF-7 and MCF-7/Dox, respectively. *t*_1_ and *t*_2_ are the average of total spectral counts of all proteins in three replicates for MCF-7 and MCF-7/Dox, respectively, and *f* is a correction factor set to 1.25.

The localizations of the identified proteins were classified as secreted, predicted secreted, plasma membrane and intracellular. Every protein was assigned a most possible location. To designate proteins as secreted proteins, the subcellular information was obtained from the PIPE (http://pipe.systemsbiology.net/) and UniProtKB. The predicted secreted proteins were predicted by SignalP 3.0 (http://www.cbs.dtu.dk/services/SignalP/) with D-score higher than 0.43 and a higher hidden Markov matrix score than 0.9 [72], [73] and SecretomeP 2.0 (http://www.cbs.dtu.dk/services/SecretomeP/) with NNscore higher than 0.5 [74]. The subcellular information of remained proteins was obtained from the PIPE and UniProtKB.

The information of the molecular function of the selected proteins was obtained from Gene Ontology. The canonical pathways of the selected DESPs were analyzed with software IPA.

### Real-time quantitative reverse transcription-PCR

Total RNAs were extracted from cultured cells using a RNeasy mini kit according to the manufacturer's instruction (Tiangen, Beijing, China). Primers for real-time PCR were designed using Primer Express software version 2.0 (Applied Biosystems, Foster City, California, USA) and synthesized by Invitrogen (Invitrogen, Shanghai, China). The primer sequences for IL-18 are 5′-CAAGGAAATCGGCCTCTATTTG-3′ (forward) and 5′-GCCATACCTCTAGGCTGGCTAT-3′ (reverse). The primer sequences for internal control glyceraldehyde-3-phosphate dehydrogenase (GAPDH) are 5′-CATGAGAAGTATGACAACAGCCT-3′ (forward) and 5′-AGTCCTTCCACGATACCAAAGT-3′ (reverse). Real-time quantitative PCR was done in an Applied Biosystems 7300 Real-Time PCR System (Applied Biosystems, Foster City, California, USA) using SYBR Green diction according to the manufacturer's instruction. The amount of IL-18 mRNA was normalized against the expression of GAPDH.

### ELISA

The concentrations of IL-18 in the cell lines and clinical samples were determined by using a commercial ELISA kit according to the manufacturer's instruction (MBL, Nagoya, Japan). To prepare the CM, 1×10^6^ cells were seeded on 10 cm dishes. When cells were grown to approximately 75% confluence, cells were further cultured in 7.5 mL serum free medium for 24 h and the CM was collected as described above. To prepare the cell lysates, cells were collected and lysed in lysis buffer (50 mM Tris-HCl, 100 mM NaCl, 1% NP40, protease inhibitor cocktail, pH 7.4). To prepare the breast cancer tissue lysates, the tissues were homogenized and lysed in lysis buffer. The lysates were then centrifuged at 13,000 rpm for 60 min, the supernatant was collected and stored at −80°C. The protein concentrations of cell/tissue lysates were determined by Bicinchoninic Acid (BCA) assay.

### Cell Viability Assay

To test the survival rate of MCF-7 in the presence of rIL-18, 1.0×10^5^ MCF-7 cells were seeded on 3.5 cm dishes. After 24 h incubation, cells were treated with different doses of doxorubicin in the presence or absence of 40 ng/mL rIL-18 (ProSpec-Tany TechnoGene, Rehovot, Israel). Three parallel samples were repeated for each concentration point of doxorubicin. After 72 h incubation, the cells were digested and viable cells were counted by trypan blue exclusion (PBS, 0.4% trypan blue) with a blood cell count board. Either supplied with rIL-18 or not, the cell survival rate was calculated by the ratio of cell numbers at different doses of doxorubicin to that in the absence of doxorubicin. The IC_50_ was calculated by fitted equation using OriginPro 8 software. To test the effect of anti-IL-18 neutralization on the survival rate of MCF-7/Dox, 1.0×10^5^ MCF-7/Dox cells were seeded on 3.5 cm dishes. After 24 h incubation, cells were treated with different doses of doxorubicin in the presence or absence of 20 or 50 ng/mL anti-IL-18 antibody (MBL, Nagoya, Japan). After 96 h incubation, the cells were digested and viable cells were counted by trypan blue exclusion with a blood cell count board. Either with or without anti-IL-18 antibody, the cell survival rate was calculated by the ratio of cell numbers at different doses of doxorubicin to that of 200 ng/mL doxorubicin. All the cell viability experiments were repeated three times as indicated using triplicate samples for cell counting analyses. Data were evaluated using the Student's *t*-test and were considered significant when p<0.05.

## Supporting Information

Figure S1 **The drug resistant characteristic of MCF-7/Dox (A) and MCF-7/DoxH (B).** The cell survival rates of each cell line at different concentrations of doxorubicin were determined and presented as mean ± SD (n = 3).
(TIF)

Figure S2 **The validation of the role of IL-18 in doxorubicin resistance.** (A) The curves of doxorubicin dose dependent survival rate of MCF-7 in the absence or presence of 40 ng/mL rIL-18. Data were presented as mean ± SD (n = 3). **, p<0.01. (B) The effect of anti-IL-18 neutralization on the survival rate of MCF-7/Dox. Data were presented as mean ± SD (n = 3). *, p<0.05, **, p<0.01.
(TIF)

Table S1 **The detailed information about the 89 DESPs between MCF-7 and MCF-7/Dox.**
(XLS)

Table S2 **The information of breast cancer patients.**
(XLS)

Text S1 **The method of the drug resistant characteristic assay of MCF-7/Dox and MCF-7/DoxH.**
(DOC)
