# Supplementary figures and images for: Discovery of IL-18 As a Novel Secreted Protein Contributing to Doxorubicin Resistance by Comparative Secretome Analysis of MCF-7 and MCF-7/Dox
Source: PLoS One. 2011 Sep 8;6(9):e24684. doi: 10.1371/journal.pone.0024684 (PMC3169632; doi:10.1371/journal.pone.0024684)

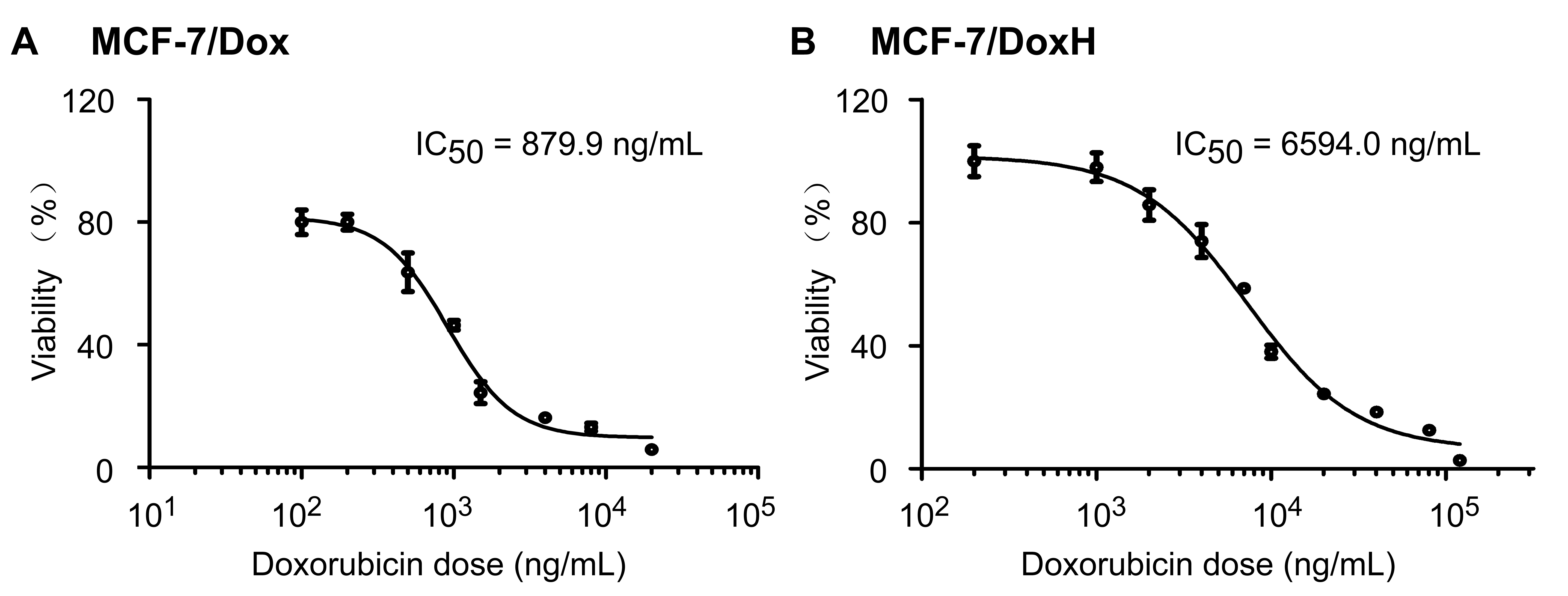

Supplement: Figure S1 — The drug resistant characteristic of MCF-7/Dox (A) and MCF-7/DoxH (B). The cell survival rates of each cell line at different concentrations of doxorubicin were determined and presented as mean ± SD (n = 3). (TIF) [file pone.0024684.s001.tif]

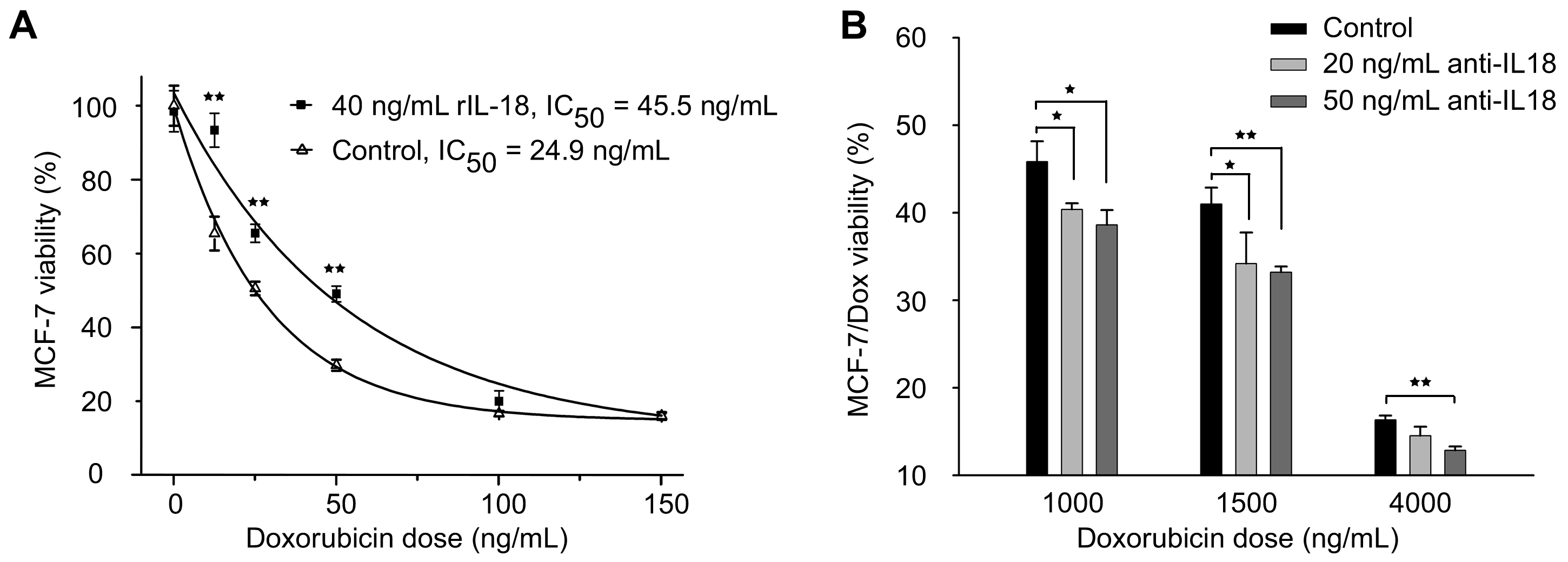

Supplement: Figure S2 — The validation of the role of IL-18 in doxorubicin resistance. (A) The curves of doxorubicin dose dependent survival rate of MCF-7 in the absence or presence of 40 ng/mL rIL-18. Data were presented as mean ± SD (n = 3). **, p<0.01. (B) The effect of anti-IL-18 neutralization on the survival rate of MCF-7/Dox. Data were presented as mean ± SD (n = 3). *, p<0.05, **, p<0.01. (TIF) [file pone.0024684.s002.tif]
