# Supplementary material for: Discovery of IL-18 As a Novel Secreted Protein Contributing to Doxorubicin Resistance by Comparative Secretome Analysis of MCF-7 and MCF-7/Dox
Source: PLoS One. 2011 Sep 8;6(9):e24684. doi: 10.1371/journal.pone.0024684 (PMC3169632; doi:10.1371/journal.pone.0024684)
Supplement: Text S1 — The method of the drug resistant characteristic assay of MCF-7/Dox and MCF-7/DoxH. (DOC) [file pone.0024684.s005.doc]

**The method of the drug resistant characteristic assay of MCF-7/Dox and MCF-7/DoxH :**

To determine the drug resistant characteristic of MCF-7/Dox and MCF-7/DoxH, for each cell line, 1.0 × 10^5^ cells were seeded on 3.5 cm dishes. After 24 h incubation, cells were treated with different doses of doxorubicin. Three parallel samples were repeated for each concentration point of doxorubicin. After 72 h incubation, the cells were trypsinized and viable cells were counted by trypan blue exclusion (PBS, 0.4% trypan blue) with a blood cell count board. The cell survival rate was calculated by the ratio of cell numbers at different doses of doxorubicin to that in the absence of doxorubicin. The curves of dose dependent cell survival rate and IC_50_ were analyzed by GraphPad Prism 5.
